# Machine learning prediction of postoperative acute kidney injury in aortic dissection patients using dynamic inflammatory markers and clinical features

**DOI:** 10.1186/s12911-026-03443-y

**Published:** 2026-03-25

**Authors:** Yansong Xu, Chunyan Huang, Yuewu Wang, Chanyu Huang, Yuan Xie, Guanbiao Liang, Caiying Li, Ruiying Wei, Junting Liu

**Affiliations:** 1 Emergency Trauma Center, The First Affiliated Hospital of Guangxi Medical University, Nanning, China; 2 Cardiothoracic Surgery, The First Affiliated Hospital of Guangxi Medical University, Nanning, China; 3 Organization and Personnel Department, The First Affiliated Hospital of Guangxi Medical University, Nanning, China

**Keywords:** Aortic dissection, Acute kidney injury, Neutrophil-to-lymphocyte ratio, Predictive model

## Abstract

**Objective:**

To develop and validate a predictive model for acute kidney injury (AKI) after aortic dissection (AD) repair by integrating the dynamic neutrophil-to-lymphocyte ratio (ΔNLR) with key clinical variables.

**Methods:**

This retrospective cohort study included 720 patients who underwent AD surgery. Patients were randomly split into training (70%) and validation (30%) cohorts. AKI was defined per RIFLE criteria. Least absolute shrinkage and selection operator (LASSO) regression was used for variable selection from demographics, medical history, imaging, surgical data, and inflammatory ratios (including preoperative, postoperative, and Δ values). Multivariable logistic regression built the final model, evaluated by discrimination (C-statistic), calibration (plots, Hosmer-Lemeshow test), and clinical utility (decision curve analysis).

**Results:**

The incidence of postoperative AKI was 17.2%. The final model incorporated four independent predictors: ΔNLR (Odds Ratio [OR]: 2.23), preoperative platelet-to-fibrinogen ratio (PFR) (OR: 1.95), open surgery (OR: 5.37), and drinking history (OR: 1.72). The model demonstrated good and consistent discrimination, with a C-statistic of 0.751 (95% CI: 0.708–0.794, *p* < 0.001) in the training cohort and 0.732 (95% CI: 0.673–0.791, *p* < 0.001) in the validation cohort. Calibration curves showed excellent agreement between predicted and observed probabilities (Hosmer-Lemeshow test *p* = 0.172). Decision curve analysis confirmed significant clinical net benefit across a clinically relevant range of risk thresholds (approximately 5% to 80%).

**Conclusion:**

We developed a robust predictive model for AKI after AD surgery, highlighting the critical value of dynamic inflammation monitoring via ΔNLR. This practical tool facilitates early identification of high-risk patients, potentially enabling timely preventive strategies to improve postoperative outcomes. External validation is warranted to confirm generalizability.

**Clinical trial number:**

Not applicable.

## Introduction

Systemic inflammation plays a central role in the progression of numerous diseases. The neutrophil-to-lymphocyte ratio (NLR), as an easily accessible inflammatory biomarker, has demonstrated significant value in assessing prognosis across various conditions [1, 2]. In the field of vascular surgery, NLR has been particularly shown to be closely associated with the risk of postoperative complications [3, 4]. This is critically important in the context of aortic dissection (AD), as the surgical intervention itself triggers a significant inflammatory response, and postoperative acute kidney injury (AKI) is a common and serious complication that markedly increases patient mortality.

In recent years, research focus has shifted from static NLR values to its dynamic changes. Dynamic NLR has proven to more accurately reflect the evolution of the systemic inflammatory state and outperforms single measurements in predicting organ failure in other diseases, such as critical COVID-19 [1, 2]. Preliminary evidence also suggests that perioperative dynamic NLR may predict AKI following cardiac surgery [3]. Given the central role of inflammation in the pathogenesis of AD [5, 6], dynamic NLR holds inherent potential for predicting AKI after AD surgery.

However, the application of dynamic NLR in this specific context remains underexplored. Therefore, this study aims to systematically evaluate the value of dynamic NLR in predicting acute kidney failure following aortic dissection surgery, with the goal of providing a simple and effective tool for the early identification of high-risk patients and improving clinical outcomes.

## Materials and methods

### Study patients

**Fig. 1:**
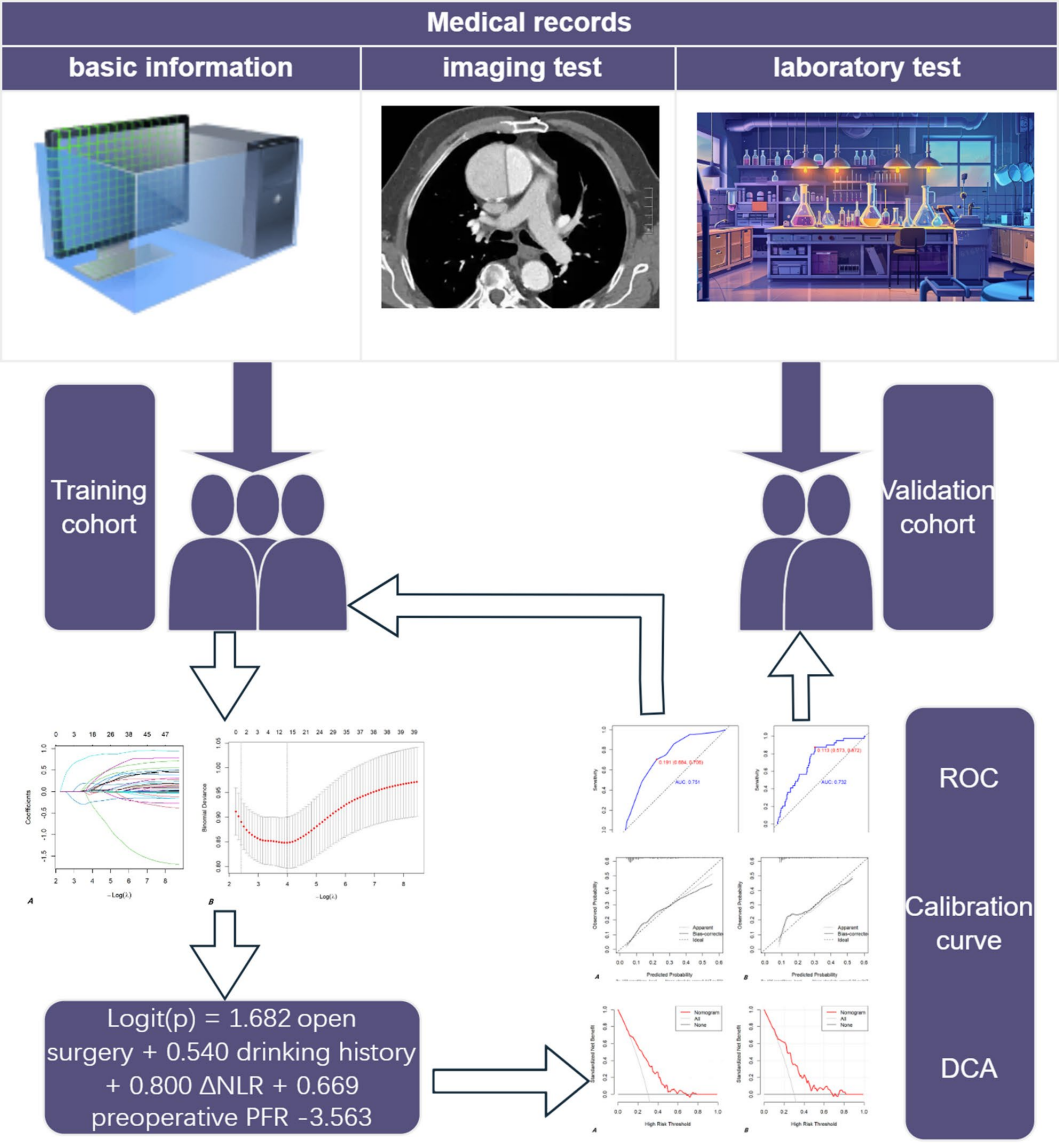
Study overview study workflow. 720 patients were randomly divided into training cohort (*n* = 503) and validation cohort (*n* = 217)

A total of 720 AD patients who received aortic surgery in the cardiovascular surgery department of First Affiliated Hospital of Guangxi Medical University from January 2011 to December 2024 were enrolled in this study. The primary end point was AKI. The parameters include: 1. Basic Information: age, gender, BMI; 2. Medical history: the time from symptom onset to admission, Heart rate, Systolic blood pressure, Diastolic blood pressure, smoking history, hypertension, diabetes, drinking history, Marfan syndrome; 3. Imaging examination: Stanford classification, renal hypoperfusion, preoperative renal artery involvement, preoperative left ventricular ejection fraction (LVEF), Maximum aortic diameter, Diameter of the false cavity, Maximum diameter of endometrial rupture; 4. Laboratory test: blood routine, blood biochemistry; 5. surgical operation have been collected. All patients undergo surgical procedures (open or minimally invasive) and blood was collected for laboratory examination on admission and with 24 h after the operation. All enrolled patients were divided into an ARF group and a non-ARF group according to whether ARF occurred within 48 h after the operation. All patients were randomly divided into a training cohort and a validation cohort according to a 7:3 ratio (Fig. 1).

### Surgical intervention was dictated by the Stanford classification of the aortic dissection

For Stanford type A dissections: All patients underwent open surgical repair via median sternotomy. This involved resection of the dissected ascending aorta with or without hemiarch or total arch replacement, using cardiopulmonary bypass (CPB) and systemic heparinization. Myocardial protection was achieved with cardioplegic arrest. In cases requiring an open distal anastomosis or arch vessel reimplantation, deep or moderate hypothermic circulatory arrest with or without selective antegrade cerebral perfusion was employed.

For Stanford type B dissections: The primary treatment strategy was thoracic endovascular aortic repair (TEVAR), considered a minimally invasive procedure and categorized as “non-open” surgery in our analysis. This was performed via femoral or iliac arterial access without the need for CPB. A subset of patients with Stanford type B dissection underwent open surgery due to specific anatomical complexities (e.g., extensive arch involvement, connective tissue disorders like Marfan syndrome, or failed prior endovascular repair). These open procedures typically involved left thoracotomy for open descending or thoracoabdominal aortic replacement. In these complex open cases, surgical adjuncts such as left heart bypass (atriofemoral bypass) or partial CPB were often utilized for distal aortic perfusion and spinal cord protection.

### Inclusion and exclusion criteria

Inclusion criteria: All patients, aged between 18 and 70 years old, underwent AD surgery procedure. All postoperative patients underwent routine blood tests and biochemical examinations on the first day after surgery. Exclusion criteria: Patients preoperative diagnosed with chronic kidney disease, congestive heart failure (left ventricular ejection fraction is less than 30%), traumatic AD, and postoperative hospitalization time < 48 h.

### Disease definition and parameter processing

All patients were diagnosed as AD by aortic CT, and aortic CT showed the rupture of aorta intima and the infiltration of contrast media. Renal artery involvement refers to the involvement of the renal artery by aortic dissection. AKI is a binary question and is defined an increase of ≥ 1.5 times in the serum creatinine or a ≥ 25% decrease in the glomerular filtration rate according to the RIFLE criteria in the IRAD database [7]. Renal hypoperfusion was assessed based on preoperative contrast-enhanced computed tomography angiography (CTA) findings. It was defined as asymmetric renal parenchymal contrast enhancement or delayed enhancement, resulting from either static obstruction (e.g., dissection flap extending into the renal artery orifice) or dynamic obstruction (e.g., true lumen compression by the false lumen) affecting one or both renal arteries. All CTA images were independently reviewed by two experienced vascular radiologists, and any discrepancies were resolved by consensus. For variables with more than 20% missing data, the corresponding records were excluded to minimize the risk of introducing potential bias that could negatively impact the model’s performance, otherwise multiple difference compensation method should be used. PLR: Platelets/lymphocytes ratio; NLR: Neutrophils/ lymphocytes ratio; PNR: Platelets/Neutrophils ratio; PWR: Platelets/ white blood count ratio; PFR: Platelet/fibrinogen ratio; DLR: D-dimer/lymphocyte ratio; Δ represents the ratio of preoperative and postoperative difference.

### The optimal cutoff values for the inflammatory ratios

The optimal cutoff values for all inflammatory ratios were derived from receiver operating characteristic (ROC) curve analysis, with the maximum Youden’s index (sensitivity + specificity − 1) serving as the selection criterion. The resulting cutoff values, along with their corresponding sensitivity and specificity, are presented in the Results section (Table 2).

### Statistical analysis

All statistical analyses were conducted using R studio (version 4.5.1). A of < 0.05 was considered statistically significant, with all p-values being two-sided. Normally distributed continuous variables were compared using the Wilcoxon rank-sum test and described as mean with standard deviation. Non-normally distributed continuous variables were tested with Mood’s median test and shown as median with interquartile ranges. Categorical variables were assessed by the λ^2^ test or Fisher exact test where appropriate. To select the most predictive variables from a large set of candidate features. Firstly, The LASSO regression was used for feature selection in the training set, and the optimal regularization parameter was selected based on the l value at one standard error (λ.1se). Then, the variables selected by LASSO are included in the multiple logistic regression model, and the forward method is used for further variable selection to establish the final prediction model. The trilinear table is completed using the Table 1 package in R language. For predictive modeling, logistic regression was implemented with the “glmnet” package, while the “rms” package was employed to conduct multivariate binary logistic regression, and plot calibration curves. The discriminatory ability of the model was evaluated using receiver operating characteristic (ROC) curves, with optimal cutoff values determined via the “pROC” package. Additionally, decision curve analysis was carried out using the “rmda” package.

**Table 1:** Summary descriptives table by groups of training and validation cohort

Parameters	Total(*n* = 720)	Training cohort(*n* = 503)	Validation cohort(*n* = 217)	*p*
Gender: Male/Female	598 / 122(83.1%)	421 / 82(83.7%)	177 /40(81.6%)	0.554
Age, mean (sd)	52.8 (11.2)	52.7 (11.1)	53.2 (11.2)	0.551
Symptom onset to admission: Acute/subacute/chronic	565 /81 /74	395 /50 /58	170 /31 /16	0.078
Emergency surgery: NO/YES	606 /114(84.2%)	429 /74(85.3%)	177 /40(81.6%)	0.253
Open surgery: NO/YES	424 /296(58.9%)	300 /203(59.6%)	124 93(57.1%)	0.587
Chest pain: NO/YES	268 /452(37.2%)	186 317(37.0%)	82 /135(37.8%)	0.903
History of hypertension: NO/YES	199 /521(27.6%)	149 /354(29.6%)	50 /167(23.0%)	0.085
Smoking history: NO/YES	347 /373(48.2%)	243 /260(48.3%)	104 /113(47.9%)	0.989
Drinking history: NO/YES	379 /341(52.6%)	260 /243(51.7%)	119 /98(54.8%)	0.487
Diabetes: NO/YES	690 /30(95.8%)	484 /19(96.2%)	206 /11(94.9%)	0.553
Heart rate, mean (sd)	84.6 (16.2)	85.1 (16.0)	83.5 (16.5)	0.242
Systolic blood pressure, mean (sd)	148 (25.8)	148 (25.5)	150 (26.3)	0.331
Diastolic blood pressure, mean (sd)	85.2 (16.6)	85.4 (16.9)	84.9 (16.1)	0.710
BMI, mean (sd)	25.0 (3.94)	25.0 (3.92)	25.2 (3.99)	0.606
Ejection fraction, mean (sd)	66.1 (8.14)	66.0 (8.30)	66.3 (7.76)	0.586
Stanford type: A/B	275 /445(38.2%)	190 /313(37.8%)	85 /132(39.2%)	0.787
Maximum aortic diameter, mean (sd)	4.32 (1.30)	4.33 (1.33)	4.31 (1.25)	0.871
Diameter of the false cavity, mean (sd)	2.32 (1.25)	2.27 (1.21)	2.43 (1.33)	0.143
Maximum diameter of endometrial rupture, mean (sd)	1.20 (0.69)	1.20 (0.72)	1.20 (0.64)	0.931
Renal artery involvement: NO/YES	441 /279(61.3%)	305 /198(60.6%)	136 /81(62.7%)	0.666
Marfan syndrome: NO/YES	696 /24(96.7%)	487 /16(96.8%)	209 /8(96.3%)	0.904
Renal hypoperfusion: NO/TES	627 /93(87.1%)	437 /66(86.9%)	190 /27(87.6%)	0.898
Preoperative PLR: ≤170 />170	612 /108(85.0%)	503 /0(100%)	109 /108(50.2%)	<0.001
Preoperative PNR: ≤27 />27	347 (48.2%)	252 (50.1%)	95 (43.8%)	0.140
Preoperative NLR: ≤6 />6	365 /355(50.7%)	255 /248(50.7%)	110 /107(50.7%)	1.000
Preoperative PWR: ≤20 />20	343 /377(47.6%)	247 /256(49.1%)	96 /121(44.2%)	0.263
Preoperative DLR: ≤909 />909	335 /385(46.5%)	231 /272(45.9%)	104 /113(47.9%)	0.680
Preoperative PFR: ≤36 />36	153 /567(21.2%)	110 /393(21.9%)	43 /174(19.8%)	0.604
PT, mean (sd)	11.9 (1.28)	11.9 (1.27)	11.9 (1.31)	0.948
FIB, mean (sd)	4.37 (1.55)	4.39 (1.57)	4.33 (1.48)	0.639
APTT, mean (sd)	31.4 (3.61)	31.3 (3.72)	31.7 (3.34)	0.158
D-dmier, median (Q1, Q3)	1129 (599,2834)	1190 (615,2847)	1079 (547,2785)	0.095
ALB, mean (sd)	37.9 (4.78)	38.0 (4.85)	37.7 (4.63)	0.465
Postoperative PLR: ≤219 />219	341 /379(47.4%)	251 /252(49.9%)	90 /127(41.5%)	0.046
Postoperative PNR: ≤18.3 />18.3	353 /367(49.0%)	252 /251(50.1%)	101 /116(46.5%)	0.427
Postoperative NLR: ≤12 />12	342 /378(47.5%)	249 /254(49.5%)	93 /124(42.9%)	0.119
Postoperative PWR: ≤16 />16	374 /346(51.9%)	264 /239(52.5%)	110 /107(50.7%)	0.718
ΔPLR: ≤76 />76	261 /459(36.2%)	177 /326(35.2%)	84 /133(38.7%)	0.414
ΔPNR: ≤13 >13	264 /456(36.7%)	182 /321(36.2%)	82 /135(37.8%)	0.745
ΔNLR: ≤10 />10	520 /200(72.2%)	367 /136(73.0%)	153 /64(70.5%)	0.559
ΔPWR: ≤12.5 />12.5	241 /479(33.5%)	165 /338(32.8%)	76 /141(35.0%)	0.622
AKI: NO/YES	596 /124(82.8%)	418 /85(83.1%)	178 /39(82.0%)	0.808
Stanford Type A (*n* = 275) Open Surgery (with CPB)	275 (100%)	190 (100%)	85 (100%)	1.000
Stanford Type B (*n* = 445)				
TEVAR (Minimally Invasive)	380 (85.4%)	265 (84.7%)	115 (87.1%)	0.587
Open Surgery	65 (14.6%)	48 (15.3%)	17 (12.9%)	0.587

## Results

### Characteristics of patients

A total of 720 patients were identified and randomly divided into a training cohort and a validation cohort by a ratio of 7:3. The demographic and clinical characteristics of these patients are summarized in Table 1. In the whole population, training, and validation cohorts, the mean ages of patients were 52.8 ± 11.2, 52.7 ± 11.1 and 53.2 ± 11.2 years, respectively. Except for statistical differences in preoperative and postoperative PLR between the two groups (*P*<0.05). The training and validation cohorts were comparable in terms of demographic and clinical characteristics (*P* > 0.05) (Table 1).

### Variables screening and construction of model to predict AKI

**Fig. 2:**
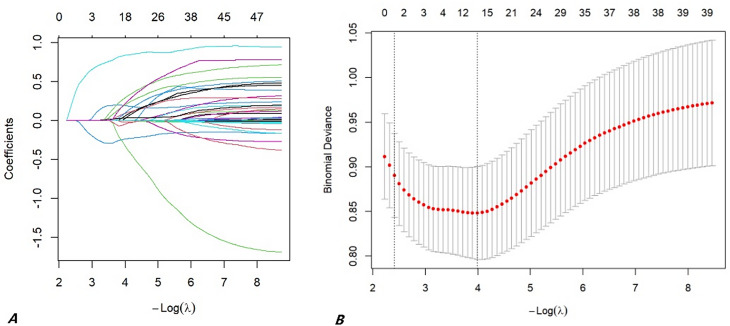
LASSO regression. (**A**) LASSO regression coefficients under diverse log λ values. (**B**) Partial likelihood deviance under diverse log λ values for LASSO

**Fig. 3:**
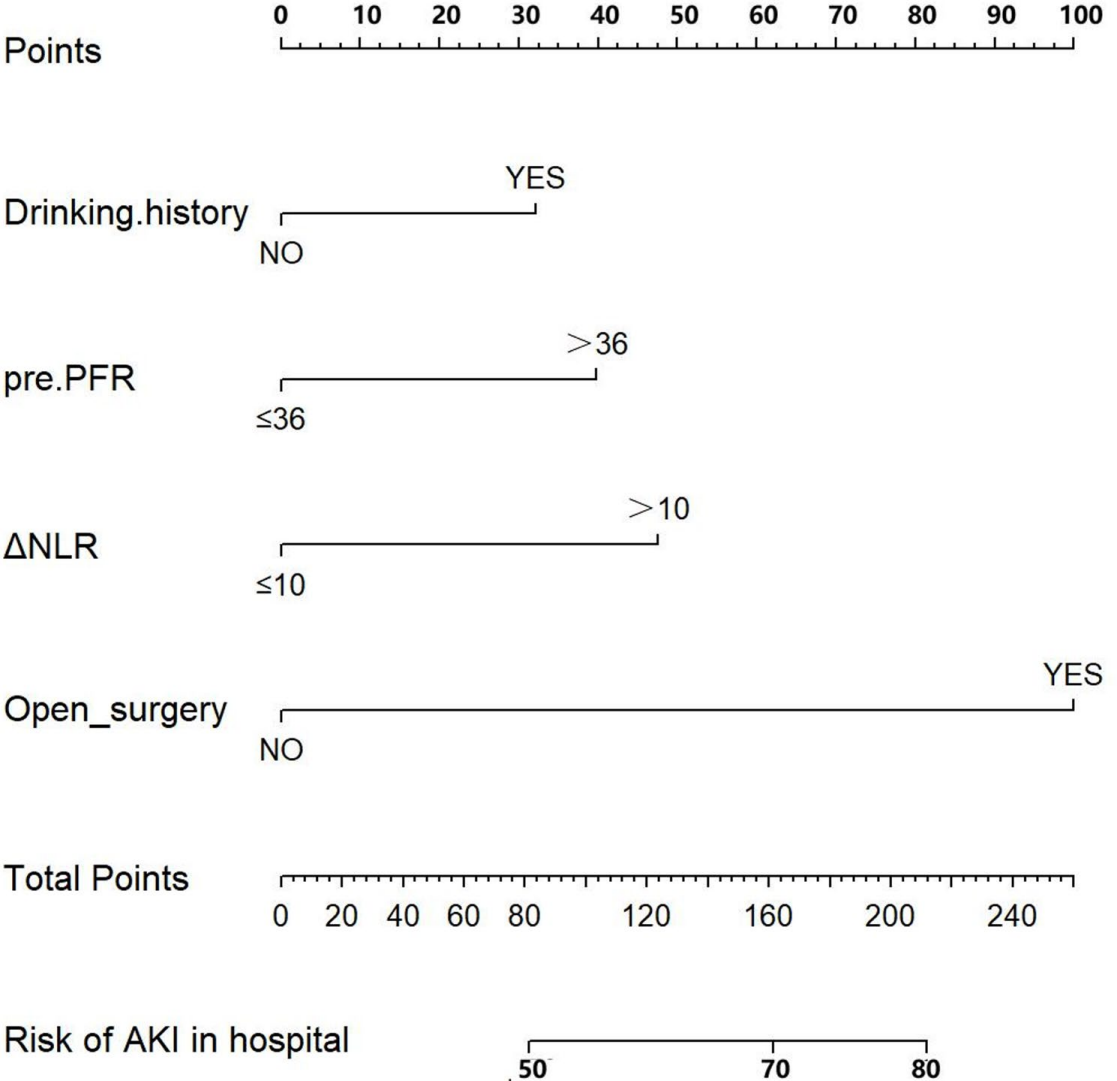
Construction of nomogram based on clinical features

The optimal cutoff values for all inflammatory ratios were derived from receiver operating characteristic (ROC) curve analysis, with the maximum Youden’s index (sensitivity + specificity − 1) serving as the selection criterion. The resulting cutoff values, along with their corresponding sensitivity and specificity, are presented in the Results section (Table 2). 503 patients were allocated in the training cohort, and 217 patients in the validation cohort. There was no statistically significant difference between the two groups of variables (Table 1). Based on the results of LASSO regression (Fig. 2), 14 variables including surgical operation: emergency surgery, open surgery; Medical history༚history of hypertension, drinking history; Imaging examination: Stanford classification, renal artery involvement, Marfan syndrome, renal hypoperfusion; Laboratory tests: FIB, APTT, preoperative PFR, preoperative NLR, postoperative PNR, ΔPNR were put in multivariable logistic regression analysis, which turned out that only the last four variables remained significant(*P* < 0.05) (Table 3). It is noteworthy that while preoperative and postoperative PLR showed distributional differences between cohorts in Table 1, they were not retained by the LASSO regression. This indicates that their predictive information for AKI was either redundant with other selected variables (e.g., captured by ΔNLR or PFR) or less robust in the multivariate context. The VIF values were all < 3, indicating that no collinearity existed between screened variables. Thus, a model to predict AKI in postoperative AD patients was developed with the four variables: Logit(p) = 1.682 ✖open surgery + 0.540✖drinking history + 0.800✖ΔNLR + 0.669✖ preoperative PFR − 3.563, in which p indicated the probability of AKI (Figure 3).

**Table 2:** ROC analysis of inflammatory ratios for predicting postoperative AKI

Inflammatory ratio	Timing	AUC	95% CI	*p*-value	Optimal cutoff	Sensitivity (%)	Specificity (%)
PLR	Preoperative	0.750	0.690–0.810	< 0.001	170	70.3	73.7
PNR	Preoperative	0.600	0.500–0.700	0.063	27	50	64.5
NLR	Preoperative	0.650	0.550–0.750	0.005	6	60.6	64.4
PWR	Preoperative	0.580	0.474–0.686	0.138	20	54.5	55.5
PFR	Preoperative	0.530	0.425–0.635	0.575	36	60.6	25.8
DLR	Preoperative	0.520	0.416–0.625	0.707	909	54.5	55.2
PLR	Postoperative	0.600	0.494–0.706	0.065	219	54.5	55.5
NLR	Postoperative	0.70	0.597–0.803	< 0.001	12	66.7	64.4
PWR	Postoperative	0.57	0.464–0.676	0.195	18.3	42.4	64.4
PNR	Postoperative	0.57	0.464–0.676	0.195	16	54.5	55.5
ΔPLR	Dynamic change	0.54	0.442–0.638	0.425	76	57.9	51.0
ΔNLR	Dynamic change	0.62	0.523–0.717	0.015	10	55.0	66.2
ΔPWR	Dynamic change	0.54	0.444–0.636	0.414	13	55.0	52.1
ΔPNR	Dynamic change	0.57	0.473–0.667	0.156	12.5	60.0	52.1

**Table 3:** Multivariate logistic regression

Variables	B	SD	Wald	df	*P*	OR	95%CI	
Open surgery	1.682	0.273	37.882	1	<0.001	5.375	3.146	9.182
Drinking history	0.54	0.258	4.376	1	0.036	1.717	1.035	2.848
ΔNLR	0.8	0.278	8.277	1	0.004	2.225	1.29	3.836
Preoperative PFR	0.669	0.34	3.866	1	0.049	1.952	1.002	3.801

### Performance and validation of the model

**Fig. 4:**
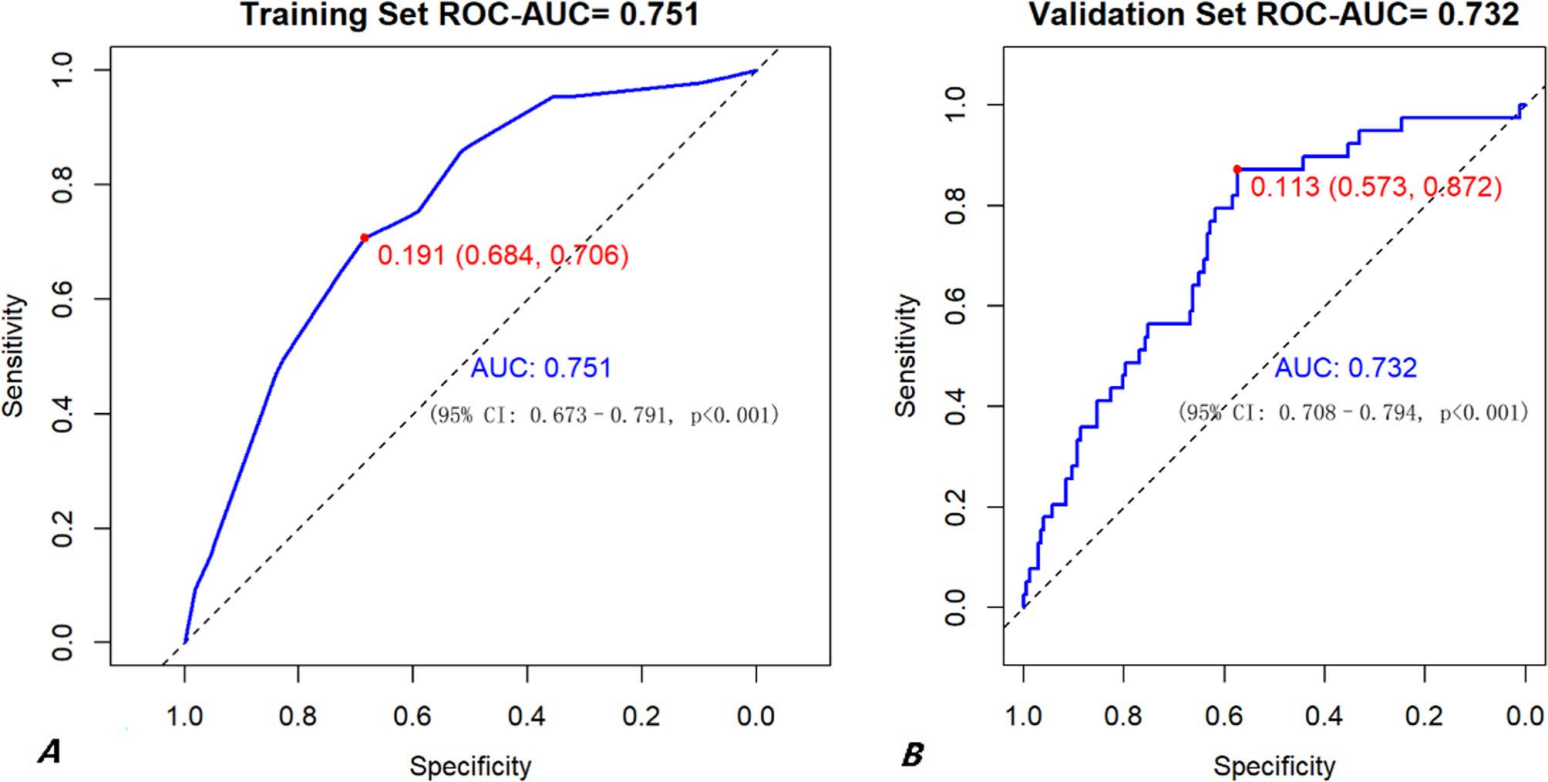
The ROC curves of the prediction model. (**A**) The ROC curve of the prediction model in training cohort. (**B**) The ROC curve of the prediction model in validation cohort

**Fig. 5:**
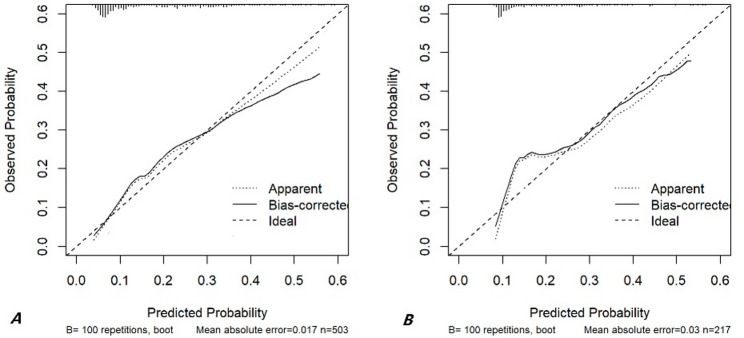
Calibration curve analysis. (**A**) Calibration curve analysis in the training cohort; (**B**) Calibration curve analysis in the internal validation cohort

Figure 4 shows the discriminative performance of the prognostic model for risk of AKI in the training and validation cohorts. The discriminative performance of the model, as indicated by the C-statistic (AUC), was 0.751 (95% CI: 0.708–0.794, *p* < 0.001) in the development set, and 0.732 (95% CI: 0.673–0.791, *p* < 0.001) in the internal validation set, demonstrating consistent and good discriminative ability. Calibration curves indicated good agreement between predicted and observed AKI probabilities in both cohorts (Fig. 5). The Hosmer–Lemeshow test yielded a non-significant result (X-squared = 9.0264, df = 6, p-value = 0.1721), supporting the model’s goodness-of-fit. Overall, the nomogram exhibited robust discrimination and calibration.

### Clinical utility of the prediction model

**Fig. 6:**
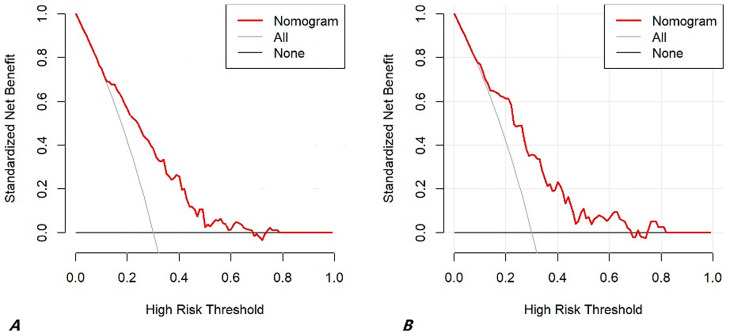
Decision curve analysis of the prediction model. (**A**) The training cohort; (**B**) The validation cohort

The clinical decision curve analysis demonstrates that the constructed nomogram has favorable clinical utility for predicting the risk of postoperative AKI in patients with aortic dissection. In both the training and validation cohorts, the curve representing the prediction model is consistently higher than the two reference lines (“All” and “None”) across a threshold probability range of approximately 5% to 80%. This indicates that, within this clinically relevant spectrum, using the prediction model for risk assessment provides a greater net benefit compared to the alternative strategies of either “intervening for all patients” or “intervening for no patients.“(Fig. 6).

## Discussion

In this large retrospective cohort study, we successfully developed and internally validated a novel predictive model integrating dynamic inflammatory indices and clinical variables to forecast acute kidney injury (AKI) following aortic dissection (AD) repair. The model demonstrated robust discrimination (C-statistic: 0.751, 95% CI: 0.708–0.794, *p* < 0.001 in the training cohort; 0.732, 95% CI: 0.673–0.791, *p* < 0.001 in the validation cohort), satisfactory calibration, and compelling clinical utility. Our central finding identifies the dynamic neutrophil-to-lymphocyte ratio (ΔNLR) as an independent predictor of postoperative AKI, providing fresh clinical insights into the inflammatory mechanisms underpinning renal injury after AD surgery. Similarly, the present researches [8, 9]. reported that 15.6–16.8% of patients with AKI required postoperative renal replacement therapy, which was close to the 17.2% reported in our study.

Our findings robustly endorse the superiority of “dynamic inflammation monitoring” over single-time-point measurements. This concept resonates with the work of Moisa et al. in critically ill COVID-19 patients, where dynamic NLR outperformed static indices in predicting the need for invasive mechanical ventilation and mortality (AUC 0.78–0.82) [2]. Within cardiovascular surgery, Usta and Abanoz reported that ΔNLR predicted AKI after coronary artery bypass grafting with an AUC of 0.848 [3]. Our study effectively translates this concept to the more complex scenario of AD repair, substantiating that the trajectory of the postoperative inflammatory response—rather than isolated pre- or post-operative values—more accurately reflects the magnitude of ongoing tissue injury and systemic inflammatory response syndrome (SIRS), thereby offering a more reliable early warning of impending organ dysfunction.

The pathogenesis of AKI post-AD surgery is multifactorial, with inflammation serving as a common thread. Primarily, the dissection itself and the surgical insult trigger a potent innate immune activation. Neutrophils, as first responders, surge in number and infiltrate tissues, directly damaging renal tubular epithelial cells via the release of reactive oxygen species, proteases, and inflammatory cytokines [5, 6]. Furthermore, neutrophils exacerbate microvascular thrombosis and renal ischemia through mechanisms like neutrophil extracellular trap (NET) formation [10]. Conversely, postoperative lymphopenia is considered a marker of a stress-induced immunosuppressive state, compromising the body’s immunomodulatory capacity. Consequently, the NLR (and ΔNLR), as a composite index, simultaneously captures both the heightened “pro-inflammatory assault” and the weakened “immunological defense.” Recent foundational research has further illuminated the central role of the NLRP3 inflammasome in AD pathogenesis [11–13]. Wu et al. demonstrated that in AD models, NLRP3 inflammasome activation leads to caspase-1-dependent degradation of contractile proteins and phenotypic switching of vascular smooth muscle cells (VSMCs), thereby weakening the aortic wall [10]. We postulate that this same inflammasome activation pathway may be triggered during renal ischemia-reperfusion injury, leading to the release of mature inflammatory cytokines like IL-1β, directly contributing to the pathology of AKI [14]. Thus, the dynamic changes in ΔNLR may indirectly reflect the activation level of inflammatory platforms like the NLRP3 inflammasome in vivo.

The final model incorporated four variables: ΔNLR, preoperative platelet-to-fibrinogen ratio (PFR), open surgery, and a history of alcohol use. Open surgery is a well-established high-risk factor, associated with more pronounced hemodilution, inflammatory activation, and hemodynamic instability [9, 15]. In our final model, ‘open surgery’ emerged as the strongest predictor of postoperative AKI (OR: 5.37). This variable encapsulates a high-risk surgical profile. For Stanford type A dissections, it represents the substantial physiologic insult of median sternotomy, cardiopulmonary bypass (CPB), potential circulatory arrest, and the associated systemic inflammatory response syndrome, hemodilution, and embolic risk—all established contributors to renal injury. For the minority of Stanford type B patients who underwent open surgery, this typically involved major thoracic or thoracoabdominal procedures, often requiring adjunctive perfusion techniques (e.g., left heart bypass), which carry their own inflammatory and ischemic risks. In contrast, the predominantly endovascular approach for type B dissections (categorized as ‘non-open’) avoids thoracotomy and CPB, leading to a comparatively lower incidence of AKI. Therefore, the ‘open surgery’ variable effectively serves as a composite marker for procedural complexity, invasiveness, and exposure to bypass-related pathophysiology. A history of alcohol use may increase renal susceptibility to acute injury by inducing a chronic inflammatory state and endothelial dysfunction [16]. The preoperative PFR balances coagulation status (platelets) and the acute phase inflammatory response (fibrinogen); its significance in the model suggests that a pre-operative state combining hypercoagulability and heightened inflammation creates a fertile ground for AKI [17, 18]. Our feature selection process, employing LASSO regression, prioritized ΔNLR over static preoperative or postoperative inflammatory ratios like PLR. This underscores a key finding: the dynamic change in the systemic inflammatory state holds greater prognostic value than a snapshot measurement. Our model, anchored by ΔNLR and preoperative PFR, offers a distinct perspective from traditional risk scores like EuroSCORE II. While EuroSCORE II effectively captures baseline comorbidity and procedural complexity, it does not incorporate dynamic, postoperative inflammatory markers. The significant predictive value of ΔNLR in our model suggests that the trajectory of the host inflammatory response after surgery provides incremental prognostic information beyond preoperative risk assessment. This supports the concept of ‘dynamic risk stratification’ in the perioperative period. The Decision Curve Analysis confirmed substantial clinical net benefit across a wide probability threshold range, indicating that employing this model for clinical decision-making (e.g., initiating more intensive monitoring, optimizing hemodynamic management, or considering targeted anti-inflammatory therapies in high-risk patients) would yield superior outcomes compared to universal intervention or non-intervention strategies.

Several limitations merit consideration and improvement in future. First, We frame this limitation within the context of retrospective research and emphasize that future prospective studies should mandate the collection of both SCr and UO data to apply the full KDIGO criteria, thereby improving diagnostic accuracy and model performance. Second, the extended study period (2011–2024) is both a strength, providing a large sample size, and a source of potential heterogeneity. During this time, surgical techniques (e.g., endovascular technology), perioperative management protocols (e.g., increased use of extracorporeal membrane oxygenation for cardiopulmonary support), and critical care concepts evolved. Furthermore, while the core surgical team was stable, an institutional learning curve effect over time cannot be excluded. These temporal changes represent unmeasured confounding factors that our retrospective model cannot fully adjust for. This limitation underscores that our model should be considered as derived from a ‘historical’ cohort, and its performance must be tested through external validation in contemporary, independent populations to ensure its ongoing relevance. Third, the model’s predictive performance is good (AUC > 0.73), there remains room for improvement, but the lack of external validation is the primary limitation of our study and a prerequisite before clinical implementation can be considered. Fourth, we did not account for intraoperative temperature management strategies, such as hypothermia during cardiopulmonary bypass or circulatory arrest, which may modulate inflammatory responses and renal susceptibility to injury. Future models could benefit from including temperature-related variables to enhance predictive accuracy. While we used LASSO regression for variable selection and validated the stability of our findings with bootstrap analysis, future studies with high-dimensional data may benefit from employing even more robust selection frameworks, such as the Knockoff filter, which provides formal control over the false discovery rate during feature selection [19, 20]. Future investigations should explore integrating more kidney-specific novel biomarkers, such as serum cystatin C, NGAL, or TLR-4 [21, 22], or employ machine learning algorithms to uncover deeper predictive patterns [23]. Regarding the model’s presentation, we deliberately opted for a regression formula over a nomogram. This decision was primarily driven by two factors: firstly, the model’s precision at the individual level was deemed insufficient to warrant the visual precision of a nomogram; Secondly, the regression formula facilitates easier integration into electronic health record systems for automated risk calculation, offering greater practicality and flexibility for clinical deployment. Ultimately, whether risk stratification based on this model translates into improved patient outcomes requires validation through prospective randomized controlled trials.

## Conclusion

We have developed and validated a practical predictive model, incorporating the dynamic inflammatory index ΔNLR, which effectively identifies patients at high risk for AKI following AD surgery. This study not only provides a tool for early clinical risk stratification but also deepens the mechanistic understanding of postoperative renal injury in AD from an inflammatory perspective, laying a theoretical foundation for future exploration of targeted anti-inflammatory strategies for renal protection.

## Data Availability

The dataset utilized and analyzed in the current research is accessible from the corresponding authors upon reasonable request.
